# MetAmyl: A METa-Predictor for AMYLoid Proteins

**DOI:** 10.1371/journal.pone.0079722

**Published:** 2013-11-19

**Authors:** Mathieu Emily, Anthony Talvas, Christian Delamarche

**Affiliations:** 1 Agrocampus Ouest - Applied Mathematics Department, Rennes, France; 2 Institut de Recherche Mathématique de Rennes, UMR6625 CNRS, Rennes, France; 3 Université Rennes 2, Rennes, France; 4 Université Rennes 1 - IGDR, UMR6290 CNRS, Rennes, France; Universita' di Padova, Italy

## Abstract

The aggregation of proteins or peptides in amyloid fibrils is associated with a number of clinical disorders, including Alzheimer's, Huntington's and prion diseases, medullary thyroid cancer, renal and cardiac amyloidosis. Despite extensive studies, the molecular mechanisms underlying the initiation of fibril formation remain largely unknown. Several lines of evidence revealed that short amino-acid segments (hot spots), located in amyloid precursor proteins act as seeds for fibril elongation. Therefore, hot spots are potential targets for diagnostic/therapeutic applications, and a current challenge in bioinformatics is the development of methods to accurately predict hot spots from protein sequences. In this paper, we combined existing methods into a meta-predictor for hot spots prediction, called MetAmyl for METapredictor for AMYLoid proteins. MetAmyl is based on a logistic regression model that aims at weighting predictions from a set of popular algorithms, statistically selected as being the most informative and complementary predictors. We evaluated the performances of MetAmyl through a large scale comparative study based on three independent datasets and thus demonstrated its ability to differentiate between amyloidogenic and non-amyloidogenic polypeptides. Compared to 9 other methods, MetAmyl provides significant improvement in prediction on studied datasets. We further show that MetAmyl is efficient to highlight the effect of point mutations involved in human amyloidosis, so we suggest this program should be a useful complementary tool for the diagnosis of these diseases.

## Introduction

Amyloid fibrils are protein aggregates that are insoluble and resistant to protease activity *in vivo* [1]. The formation and the accumulation of amyloid aggregates, as implicated in the cellular death process, are common features of a variety of neurodegenerative diseases such as Parkinson's, Alzheimer's, and Huntington's diseases [2], [3]. Extensive researches have shown a large number of biological mechanisms involved in amyloidogenesis. Mutations, maturation, protein synthesis errors, inappropriate proteolysis and protein environment modification might lead to the formation of amyloid fibrils [4]. Because of the complexity of amyloidogenesis, predicting the capacity for a given protein to form amyloid fibrils remains as of today a very challenging task.

A lot of studies has tried to understand the biological mechanisms implicated in amyloidosis. Thus, all amyloid fibrils are characterized by protein misfolding responsible for a common cross-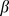 architecture [5], [6]. Moreover, recent studies highlighted the fact that amyloid formation is mainly a sequence-specific process [7]–[9] although proteins able to form amyloid-like fibrils share very little similarity in native three-dimensional structure [3], [10]. Theoretical and experimental evidence further indicate that short peptidic sequences, often called “hot spots”, play a major role in amyloidogenesis [7], [8], [11]. Hot spots can form a complementary interface with an identical segment and allow the formation of a steric zipper made by two 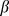 sheets that form the spine of an amyloid fibril. As the global structure of proteins is likely to modulate amyloid propensity, the length of hot spot segments might vary considerably. However, it has been experimental demonstrated that the length of six residues, corresponding to hexapeptides, is essential and sufficient for a segment to induce amyloid conversion of an entire protein domain [12], [13].

Understanding the role of short sequences in amyloid fibrilation is so crucial that the past few years have seen the development of a large number of methods dedicated to the prediction of amyloid hot spots in proteins. In 2011, Hamodrakas proposed an overview of the predictive methods and their related software published since 2004 [14]. The author provided a short description of different algorithms as for example: SALSA [15], 3D profile [16], [17], Pre-Amyl [18], PASTA [19], AGGRESCAN [20], [21], Zyggregator [22], TANGO [23], AMYLPRED [24], PAFIG [25], Net-CSSP [26], BETASCAN [27], FoldAmyloid [28], Waltz [29]. Other methods have been developed, taking into account additional features, such as amyloid fibril structural conformations, and the effect of sequence mutations [30]–[33]. At this point it should be noted that TANGO was not developed to detect amyloidogenic regions, but rather 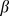-sheet aggregates that are considered as key intermediates on path to ordered fiber assembly [34], [35].

The large number of predictive methods reflects the complexity of the biological mechanisms involved in amyloidosis. It is very likely that the formation of amyloid fibrils is an intricate phenomenon in which many features interplay (secondary structures formation, disorder propensity, hydrophobicity, structural modeling energy, physico-chemical properties, amino-acid context). However, existing predictors individually account for a very few number of features, thus reducing the overall predicitive capacity of each method. The idea of associating different predictors to increase the detection power was first introduced in AMYLPRED [24], recently followed by the new version AMYLPRED2 [36]. AMYLPRED2 accounts for the diversity of 11 individual predictors by the use of a consensus. Nevertheless, the method faces a main limitation concerning the weighting process for the combined predictors that impact the accuracy of the approach. The consensus is based on binary predictions while using the prediction scores as input in a meta-prediction might increase the accuracy of the prediction.

In this paper, we propose a novel method based on a statistical approach that efficiently combines well-known existing methods into a meta-predictor in order to improve hot spot prediction. The goal of our meta-predictor, called MetAmyl for METa-predictor for AMYLoid proteins, is to exploit each individual predictor to account for a large number of features known to play a role in amyloidogenesis. The proposed approach is built on a statistical framework that aims at selecting and combining individual predictors by computing a weighted combination of predictor scores. The design of the linear combination is achieved through a logistic regression model that weights the input scores to provide the best estimate of the probability of a peptide to be an amyloidogenic segment. The estimation of our logistic model is performed on a publicy available training dataset [16], [29] and is decomposed into two main steps. In a first step, we automatically select the most informative and complementary set of individual predictors among the existing methods in the literature. The four selected predictors were SALSA, PAFIG, FoldAmyloid and Waltz. In our case, in a second step, weights are statistically assigned to each of the selected scores by maximizing a likelihood function.

The evaluation of MetAmyl is composed of two parts. In a first part, the training dataset is used to evaluate MetAmyl accuracy with respect to well-known predictors by estimating their capacity to correctly detect amyloid-forming hexapeptides as well as non-amyloid-forming hexapeptides. For this evaluation, cross-validation has been performed on the training dataset to accurately estimate the performances of MetAmyl. In a second part, we used two recently published dataset, one composed of 33 proteins from the amylome and the other made by randomly shuffle sequences from the the N-terminus of the Huntington's disease protein huntingtin, to investigate the predictive capacity of each compared predictor on experimentally-validated regions [36]. Comparative analyses show that MetAmyl improves predictions on the three datasets. Moreover, to illustrate the benefit of a prediction score instead of a binary prediction as proposed by other metapredictors, we evaluate MetAmyl prediction of the effect of mutations in human fibrinogen-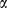. Our study points out the ability for MetAmyl to quantitatively predict the effect of mutations involved in renal amyloidosis.

## Materials and Methods

In this section, the statistical framework used to build MetAmyl score as a combination of individual predictor scores is first described. Then, MetAmyl computation together with the companion online application are presented. Finally, the three independent datasets used to train and validate MetAmyl are described as well as the comparative analysis of MetAmyl against 9 other predictors.

### MetAmyl score

MetAmyl is based on a logistic regression model that aims at weighting the different input scores to provide the most relevant combination of individual predictors. In this study, we used eleven input scores in the logistic framework. These eleven scores correspond to eleven recently published predictive algorithms: PASTA, SALSA, AGGRESCAN, PAFIG, FoldAmyloid (5 tables), TANGO and Waltz. These eleven predictors were chosen as proposing either an executable code or a sufficiently detailed algorithm to be implemented. The design of our model relies on the training dataset and is composed of two main steps, both performed using the statistical software R [37], [38]. First, we automatically select the set of individual predictors using stepwise variable selection algorithm [39]–[42]. This first step is common in supervised classification as variable selection generally alleviates the effect of the curse of dimensionality, enhances generalization by reducing overfitting and also improves model interpretability[38], [42]. In our case, training the complete model, made by the 11 individual predictors, requires the estimation of 12 coefficients. With respect to the size of the training dataset (278 sequences), training of the complete model lacks in accuracy due to high variability in coefficient estimation. As a result, four individual predictors are statistically selected in MetAmyl as being the most informative and complementary set of individual prediction scores: SALSA, PAFIG, Waltz and the first table from FoldAmyloid. One can remark that the 7 other scores were removed as being correlated with the selected individual predictors. Indeed, the five tables from FoldAmyloid are highly correlated; PASTA and SALSA showed very strong correlation; TANGO was very closed to Waltz and AGGRESCAN was correlated with FoldAmyloid.

Thus, MetAmyl is given by the following regression model:
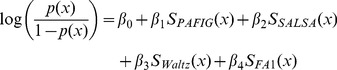
(1)where 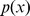 is the probability that the hexapeptide 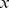 is an “hot spot”. 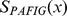, 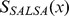, 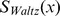 and 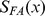 are the amyloidogenic scores for 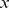 obtained with the four individual predictors PAFIG, SALSA, Waltz and the first table from FoldAmyloid. In a second step, the estimation of the regression coefficients is achieved by maximizing the likelihood of the obtained logistic model. Details of the variable selection procedure are given in Text S1 (see paragraph Stepwise variable selection in section Supplementary Methods and Table S1). Furthermore, values of the estimated 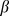 coefficients are reported in the paragraph MetAmyl Score in Text S1. The interpretation of these coefficients, that act as weights to each individual predictor, reveals that PAFIG, SALSA, Waltz and FA1 provide equivalent contribution to MetAmyl score (see paragraph Interpretation of the coefficients in section Supplementary Methods).

### Implementation, profile calculation and hot spot prediction

MetAmyl is available online at the following url: http://metamyl.genouest.org/. For an input amino-acid sequence, an amyloidogenic profile is computed by the use of a sliding window with a fix number of 6 amino acids. The score for each hexapeptide is obtained by applying MetAmyl score as described in Equation 1. The computation of the complete profile is performed by assigning the score of each hexapeptide to its third residue.

MetAmyl has been designed to manipulate large scale datasets. We first computed the 64,000,000 hexapeptides scores corresponding to the combinatorial diversity of amino-acids and we stored them on the server. Thus, the building of the profile of an input sequence consists in uploading MetAmyl scores from the server instead of calculating it for each window, which accelerates the computation of the MetAmyl profile.

If the MetAmyl score of an hexapeptide is above the best global accuracy threshold, the hexapeptide is predicted as amyloid-forming. The best global accuracy threshold is statistically obtained by maximizing a function of the sensitivity and the specificity of our meta-predictor on the training dataset. More precisely, we focused on maximizing the distance to the upper-left corner in the ROC curve, which corresponds to the cut-off that maximises the quantity 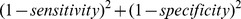 [43]. In our analysis, we obtained the same optimal cut-off by using Youden's J statistic that aims at maximizing 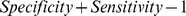 [44]. A high specificity threshold and a high sensitivity threshold are also proposed as alternatives on the MetAmyl Website, allowing users to analyze their results in different conditions. To prevent from overfitting, we used a Leave-One-Out cross-validation to estimate MetAmyl thresholds.

A “hot spot” is then defined as a contiguous serie of amyloidogenic hexapeptides. The use of a sliding window allows the detection of variable length hot spots. In order to quantify the amyloidogenicity propensity of a hot spot, the Total Area (TA) and the Normalized Hot Spot Area (NHSA) are calculated following the method used in AGGRESCAN [21]. TA is defined as the sum of scores of hexapeptides along the entire input sequence and NHSA is the area between the threshold and the profile divided by the length of the predicted segment.

### Datasets

The predictive performances of MetAmyl were compared to existing methods using the three independent datasets called respectively **training dataset**, **amylome subset** and **htt** 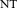.

The **training dataset** is a compilation of the Amylhex and Waltz databases designed by [29] and contains 278 hexapeptides for which experiments have been performed to determine their capacity to form amyloid aggregates. It is composed of 116 amyloid-forming hexapeptides (positive set) and 162 non-amyloid-forming hexapeptides (negative set). In our study, the training dataset was first used to estimate MetAmyl score and next to evaluate the capacity of MetAmyl to predict the amyloid status of hexapeptides.

The second dataset, called **amylome subset**, is composed of 33 proteins from the amylome and has been recently used to evaluate AMYLPRED2 predictions [36]. The **amylome subset** is a collection of proteins for which experimental data validated 70 hot spots. Details regarding the dataset and the references that support hot spots validation can be found in Table S1 of [36]. Assessing the accuracy of hot spots prediction methods is a difficult task because there is only a relatively small number of experimentally confirmed amyloid regions (true positive, TP), and even less for confirmed non-amyloid regions (true negative, TN). Another essential element involved in the difficulty to classify peptides in a positive or negative group is linked to the polymorphism of amyloid fibrils, which may depend on the experimental conditions used [45]–[47]. These are reasons why we chose the valuation dataset proposed by Tsolis *et al.*, enabling a fair comparison between AMYLPRED2 and MetAmyl which both propose consensus-based methods to predict aggregation prone regions.

In complement to the amylome subset we used a third dataset, called **htt** 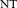, recently published by Roland and collaborators [48]. It is composed of peptides generated from the human Huntingtin protein. Using the 18 amino acids of the N terminus of the wild type protein the authors synthesized 15 scrambled sequences (peptides with the same amino acids but in a different order) whose aggregative properties were studied in vitro in simulated physiological conditions. As summarized in Table S6, 3 peptides grow rapidly into amyloid fibrils, 2 peptides aggregate more slowly and one aggregates only at high concentrations. It results that our third dataset is composed of 6 amyloid peptides and 10 non-amyloid peptides.

### Comparative analysis

Based on the training dataset, we first compared the overall performances of each predictor by the use of Receiver Operating Characteristic (ROC curves). ROC curves estimation is based on sensitivity and specificity that are estimated at different thresholds applied to the output score. Thus, ROC curve computation is not feasible for AMYLPRED2 as its prediction is binary. We further investigated the classification performances of each predictor based on confusion matrices obtained by applying provided hot spot thresholds to each score using the training dataset. Confusion matrices are summarized by four classical indicators: accuracy measured as 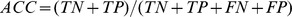, sensitivity as 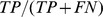, specificity as 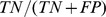 and Matthews Correlation Coefficient as 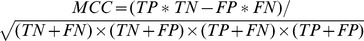, where 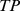 (resp. 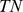, 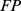, and 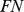) is the number of True Positives (resp. True Negatives, False Positives and False Negatives). For the following individual predictors, 3D profile, AGGRESCAN, FoldAmyloid, PAFIG, PASTA, SALSA and TANGO, we used defined thresholds as proposed by the authors. For Waltz, the *best overall performance* threshold was used. Regarding our meta-predictor, MetAmyl, we used a Leave-One-Out Cross Validation of the classification results in order to avoid overfitting. To allow a better interpretation of the observed differences between compared methods, confidence intervals for all indicators have been computed using bootstrap replicates [43], [49], [50].

We tested MetAmyl against the following individual predictors 3D profile, AGGRESCAN, FoldAmyloid, PAFIG, PASTA, SALSA, TANGO and Waltz on the amylome subset. We also compared MetAmyl performances against the metapredictor AMYLPRED2. By screening the 33 proteins of the amylome subset, we counted, for each predictor, the number of true positives (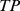), true negatives (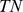), false positives (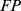) and false negatives (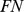) on a per residue basis, as suggested by [36]. To summarize predictor classification, we further computed the following values: sensitivity, specificity, Matthews Correlation Coefficient, Q value measured as (sensitivity+specificity)/2 and F1 score as 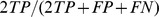. A similar comparative analysis has been conducted on the htt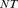 dataset.

## Results

MetAmyl aims at accounting for most biological features implicated in the amyloidogenesis by combining the asset of existing predictors. To evaluate the benefit of our approach we compared the performances of MetAmyl against 9 predictors on three independent datasets.

### Comparative analysis on the training dataset

The aim of our analysis of the training dataset was to evaluate and to compare the global accuracy of MetAmyl against existing predictors. At first, predictor performances were assessed by the use of ROC curves. Then, confusion matrices for each predictor were investigated in order to group predictive methods with respect to their statistical patterns.

**Figure 1:**
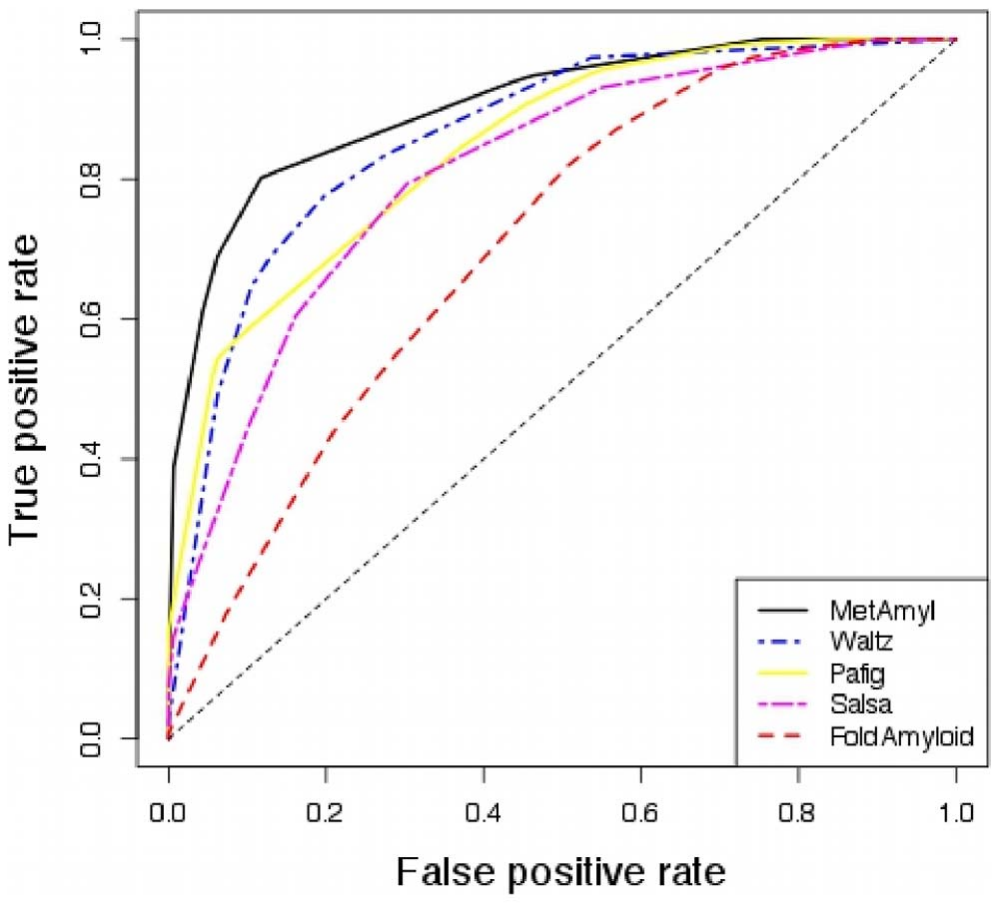
Receiver operating characteristic (ROC curves) obtained for the 4 selected predictors, PAFIG, SALSA, Fold Amyloid and Waltz, and Leave-One-Out cross validated MetAmyl on the training dataset.

Figure 1 displays the ROC curves for MetAmyl, obtained with Leave-One-Out cross-validation, and the four selected predictors: FoldAmyloid, PAFIG, SALSA and Waltz. One can see that MetAmyl outperformed the individual predictors. MetAmyl ROC curve is indeed above the other curves except in case of very high sensitivity. Similar conclusions are obtained when comparing the ROC curves for all compared predictors (see Figure S1). This result is confirmed by the comparison of the AUC (Area Under the Curve) for the different predictors. Results reported in Table 1 show that MetAmyl has the highest AUC (0.89) which is significantly higher than the other methods, according to DeLong's test (DeLong, 1988). Significance was also assessed using bootstrap replicates that allow for the estimation of 95% confidence intervals (Fawcett, 2006 and Robin, 2011) demonstrating that none of the AUC fell into MetAmyl AUC confidence interval. Furthermore, in order to perform a more useful comparison, we limited the calculation of the AUC for False Positive Rate in (FPR: 0–20%) and in (FPR: 0–5%). Results displayed in Table 1 revealed that MetAmyl has a significantly higher AUC than all other methods in case of low False Positive Rate.

**Table 1:** Area Under the Curve (AUC) based on the training dataset.

Predictor	AUC [95% CI]	p.value	AUC [95% CI]	AUC [95% CI]
		(AUC vs MetAmyl AUC)	(FPR: 0–20%)	(FPR: 0–5%)
MetAmyl	0.89 [0.87–0.92]	1	0.13 [0.12–0.15]	0.018 [0.014–0.024]
Waltz	0.85 [0.82–0.88]	0.029	0.10 [0.07–0.11]	0.005 [0.002–0.011]
PAFIG	0.82 [0.79–0.86]	0.016	0.10 [0.08–0.11]	0.011 [0.008–0.016]
PASTA	0.80 [0.77–0.84]	6.7 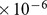	0.08 [0.07–0.10]	0.005 [0.002–0.010]
SALSA	0.79 [0.76–0.83]	8.8 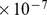	0.08 [0.06–0.09]	0.007 [0.005–0.010]
AGGRESCAN	0.76 [0.72–0.80]	2.1 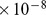	0.07 [0.05–0.08]	0.003 [0.001–0.006]
3D profile	0.75 [0.72–0.79]	1.9 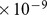	0.07 [0.06–0.09]	0.008 [0.005–0.011]
FoldAmyloid	0.69 [0.65–0.73]	1.7 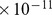	0.04 [0.03–0.05]	0.001 [0.000–0.003]
TANGO	0.67 [0.64–0.71]	2.1 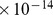	0.05 [0.03–0.06]	0.003 [0.001–0.006]

Area Under the Curve (AUC) was obtained from the ROC curves of 9 predictors: AUC cannot be computed for AMYLPRED2 as it provides only a binary prediction. For each method, the global AUC, the AUC for the False Positive Rate range of 0–20% and the AUC for the False Positive Rate range of 0–5% are reported. Numbers in brackets correspond to 95% confidence intervals (95% C.I.) that were obtained using bootstrap replicates [43]. The comparison of MetAmyl AUC and the other methods is summarized by the p.value obtained with Delong's method [49]. For the MetAmyl classifier, results were obtained using a Leave-One-Out Cross Validation.

Comparative analysis of the confusion matrices obtained for each predictor is summarized in Table 2. Accuracy, sensitivity, specificity and the Matthews Correlation Coefficient (MCC) are reported for the 10 compared predictors. 95% confidence intervals, obtained using bootstrap replicates [43], [50] are also reported. Based on results presented in Table 2, predictors are clustered in four groups according to their statistical patterns. In a first group, predictors MetAmyl and Waltz showed reasonable accuracy characterized by high sensitivity and high specificity. The second group is composed of 4 predictors (PAFIG, SALSA, AGGRESCAN, FoldAmyloid) having good sensitivity but poor specificity. On the contrary, the third group includes 3 predictors (PASTA, TANGO, AMYLPRED2) sharing an acceptable specificity and a low sensitivity. Finally the fourth group characterizes 3D profile that showed lack both in sensitivity and specificity.

**Table 2:** Prediction performances, based on the training dataset are given for the 10 compared predictors.

Predictor	ACC [95% CI]	Sensitivity [95% CI]	Specificity [95% CI]	MCC [95% CI]
MetAmyl	0.84 [0.81–0.87]	0.78 [0.73–0.82]	0.88 [0.85–0.92]	0.67 [0.60–0.72]
Waltz	0.79 [0.76–0.82]	0.73 [0.68–0.77]	0.83 [0.79–0.88]	0.57 [0.50–0.63]
PAFIG	0.69 [0.65–0.72]	0.84 [0.80–0.89]	0.57 [0.53–0.63]	0.42 [0.36–0.49]
PASTA	0.71 [0.67–0.74]	0.38 [0.32–0.44]	0.94 [0.92–0.97]	0.41 [0.34–0.47]
SALSA	0.69 [0.66–0.73]	0.84 [0.80–0.89]	0.59 [0.54–0.64]	0.43 [0.37–0.50]
AGGRESCAN	0.55 [0.51–0.59]	0.92 [0.89–0.95]	0.29 [0.24–0.34]	0.26 [0.20–0.32]
3D profile	0.66 [0.63–0.70]	0.59 [0.53–0.65]	0.71 [0.67–0.75]	0.31 [0.23–0.37]
FoldAmyloid	0.61 [0.58–0.65]	0.87 [0.83–0.91]	0.43 [0.38–0.48]	0.32 [0.26–0.39]
TANGO	0.69 [0.66–0.73]	0.52 [0.46–0.58]	0.82 [0.78–0.86]	0.36 [0.29–0.43]
AMYLPRED22	0.79 [0.76–0.82]	0.65 [0.60–0.71]	0.88 [0.85–0.92]	0.57 [0.50–0.63]

For each method, the accuracy, the sensitivity, the specificity and the Matthews correlation coefficients (MCC) are reported. Numbers in brackets correspond to 95% confidence intervals (95% C.I.) that were obtained using bootstrap replicates (Robin *et al.*, 2011). For the MetAmyl classifier, results were obtained using a Leave-One-Out Cross Validation.

In terms of accuracy, MetAmyl outperformed the other methods with a correct classification rate of 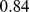. Predictors Waltz and AMYLPRED2 gave acceptable results with accuracies of 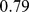. However, according to confidence intervals, Waltz and AMYLPRED2 accuracies are significantly lower than MetAmyl accuracy (see Table 2). All other methods showed poor global performances with a correct classification rate lower than 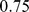. These results are enhanced by the fact that MetAmyl MCC is significantly higher than the compared predictors.

In more details, the predictors from the second group (PAFIG, SALSA, AGGRESCAN and FoldAmyloid) are able to detect amyloid hexapeptides (sensitivity higher than 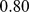). However these four predictors showed a tendency to also detect false positives with specificities lower than 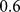. For AGGRESCAN and FoldAmyloid, the true negative rate is even lower than 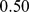 meaning that more than half of the non-amyloid hexapeptides is misclassified. AMYLPRED2, PASTA and TANGO provided an opposite statistical pattern. These three predictors have indeed a very good capacity at predicting non-amyloid hexapeptides, with specificities higher than 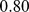. The counterpart is their poor sensitivity of their predictions.

On the training dataset, our results demonstrate that using a weighted combination of predictors increases the prediction accuracy of hexapeptides. MetAmyl accuracy is not over estimated by effect of overfitting as results presented in Tables 1 and 2 used a Leave-One-Out cross-validation. Effect of cross-validation on MetAmyl performances is displayed in Table S2 that reports the global measures (AUC, ACC and MCC) in four situations: no cross-validation, Leave-One-Out cross-validation, 10-fold cross-validation and 20-fold cross-validation. Results without cross-validation are artificially improved compared to performances in the three cross-validation set-ups that are equivalent.

### Investigation of a subset of proteins from the amylome

In a second study, we compared MetAmyl prediction with 9 predictors by estimating the ability of each method to detect hot spots in a set of 33 proteins belonging to the amylome [36]. Our results show that MetAmyl has the best Q value, MCC and F1 score compared to the other predictors (see Table 3 and Table S5 for the detail of MetAmyl hotspot prediction). Regarding Q values, MetAmyl (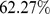) is followed by PAFIG (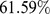), AMYLPRED2 (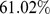) and Waltz (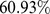). Furthermore, MetAmyl has the highest MCC (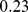) while the second and third best MCC are AMYLPRED2 (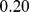) and PAFIG (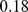). Concerning F1 score, MetAmyl (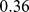) is followed by PAFIG (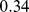), AMYLPRED2 (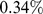) and Waltz (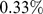). Using bootstrap replicates, we computed 95% confidence intervals and results reported in Table S3 show that MetAmyl has a significantly higher MCC and F1 score than the other methods. Moreover, MetAmyl has a significantly higher Q value than all other predictors except PAFIG.

**Table 3:** Evaluation of the performance of the tool MetAmyl on a subset of 33 proteins of the amylome.

Predictor	TP	TN	FP	FN	Sensitivity (%)	Specificity (%)	Q (%)	MCC	F1
MetAmyl	508	5519	1064	740	40.71	83.84	62.27	0.23	0.36
Waltz	710	4300	2273	548	56.43	65.42	60.93	0.16	0.33
PAFIG	651	4695	1878	607	51.75	71.43	61.59	0.18	0.34
PASTA	230	6099	484	1018	18.43	92.65	55.54	0.14	0.23
SALSA	869	3123	3460	379	69.63	47.44	58.54	0.13	0.31
AGGRESCAN	445	5210	1363	813	35.37	79.26	57.32	0.13	0.29
3D profile	224	5762	821	1024	17.95	87.53	52.74	0.06	0.20
FoldAmyloid	340	5659	924	908	27.24	85.96	56.60	0.13	0.27
TANGO	172	6282	291	1086	13.67	95.57	54.62	0.14	0.20
AMYLPRED2	478	5512	1071	770	38.30	83.73	61.02	0.20	0.34

MetAmyl is compared to 9 other methods on a subset of 33 proteins (Tsolis *et al.*, 2013).

MetAmyl performances on the amylome subset are confirmed by the comparison of the area under the ROC curves displayed in Figure S2. Numerical results reported in Table S4 shows that MetAmyl has the highest AUC (0.67) followed by PAFIG (AUC = 0.62), SALSA (AUC = 0.61) and PASTA (AUC = 0.61). Furthermore, according to DeLong's test [49], MetAmyl AUC is significantly higher than AUC for the other methods: pvalue obtained with the comparison with the second best method, PAFIG, is equal to 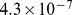. As pointed out in [36], upcoming experimental data may generate changes in results and numbers reported in Tables 3, S3 and S4, as well as Figure S2.

The study of the amylome subset shows that the four individual predictors including in MetAmyl, namely PAFIG, SALSA, Waltz and FolAmyloid, exhibit different statistical patterns. On one hand, PAFIG and Waltz are both specific with a reasonable sensitivity. On the other hand, SALSA is highly sensitive with a poor specificity and finally, FoldAmyloid has a very high specificity balanced by a very low sensitivity. Thus, MetAmyl performance on the amylome subset (independent of the training dataset) establishes that MetAmyl efficiently combines these four predictors according to their complementary statistical patterns. MetAmyl benefits from the sensibility of SALSA while accounting for the FoldAmyloid, PAFIG and Waltz specificities, allowing for a better control of the false positive rate.

### Investigation of scrambled sequences from htt
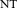


Using a recently published dataset composed of scrambled sequences from 17-amino acid peptide segment, we compared MetAmyl predictive performances with 9 existing predictors [48]. Predictions are summarized by the three following statistical quantities: Q value, MCC and F1 score (see Table 4). Furthermore, 95% confidence intervals have been computed using bootstrap replicates (see Table S7). Our comparative analysis shows that only 2 sequences over 16 are missclassified by MetAmyl which makes MetAmyl the best predictor with respect to Q value, MCC and F1 score (see Table 4). MetAmyl has indeed the best Q value (83.33%) which is statistically higher than the other predictors as for example AMYLPRED2 that have the second highest Q value (75%). Furthermore, MetAmyl has statistically the highest MCC (0.74) while the second and third best MCC are AMYLPRED2 (0.52) and Waltz (0.48). Regarding F1 score, MetAmyl reach the score of 0.8 and is followed by AMYLPRED2 (0.7) that falls into MetAmyl 95% confidence interval. The 8 other predictors have a significantly lower F1 score.

**Table 4:** Evaluation of the performance of the tool MetAmyl on scrambled sequences from the 17- amino acid N-terminal segment of the Huntingtin protein.

Predictor	TP	TN	FP	FN	Sensitivity (%)	Specificity (%)	Q (%)	MCC	F1
MetAmyl	4	10	0	2	66.67	100	83.33	0.75	0.8
Waltz	2	10	0	4	33.33	100	66.67	0.49	0.5
PAFIG	5	3	7	1	83.33	30	56.67	0.15	0.56
PASTA	5	2	8	1	83.33	20	51.67	0.04	0.53
SALSA	5	6	4	1	83.33	60	71.67	0.42	0.67
AGGRESCAN	6	1	9	0	100	10	55	0.2	0.57
3D profile	4	0	10	2	66.67	0	33.33	−0.49	0.4
FoldAmyloid	6	1	9	0	100	10	55	0.2	0.57
Tango	2	7	3	4	33.33	70	51.67	0.03	0.36
AMYLPRED2	6	5	5	0	100	50	75	0.52	0.71

MetAmyl is compared to 9 other methods on a set of 16 amino acid segments obtained from the Huntingtin protein [48].

Our results show that the 4 individual predictors used to built MetAmyl score have complementary statistical patterns. On one hand PAFIG and FoldAmyloid are highly sensitive with a very poor specificity. On the other hand, Waltz combines high specificity and low sensitivity. Finally SALSA proposes an acceptable trade-off between specificity and sensitivity. Thus, MetAmyl performance on the Huntingtin dataset proves that MetAmyl efficiently combines these four predictors by using Waltz and SALSA specificities and PAFIG, SALSA and FoldAmyloid sensitivities.

### Example of disease-associated variants

**Figure 2:**
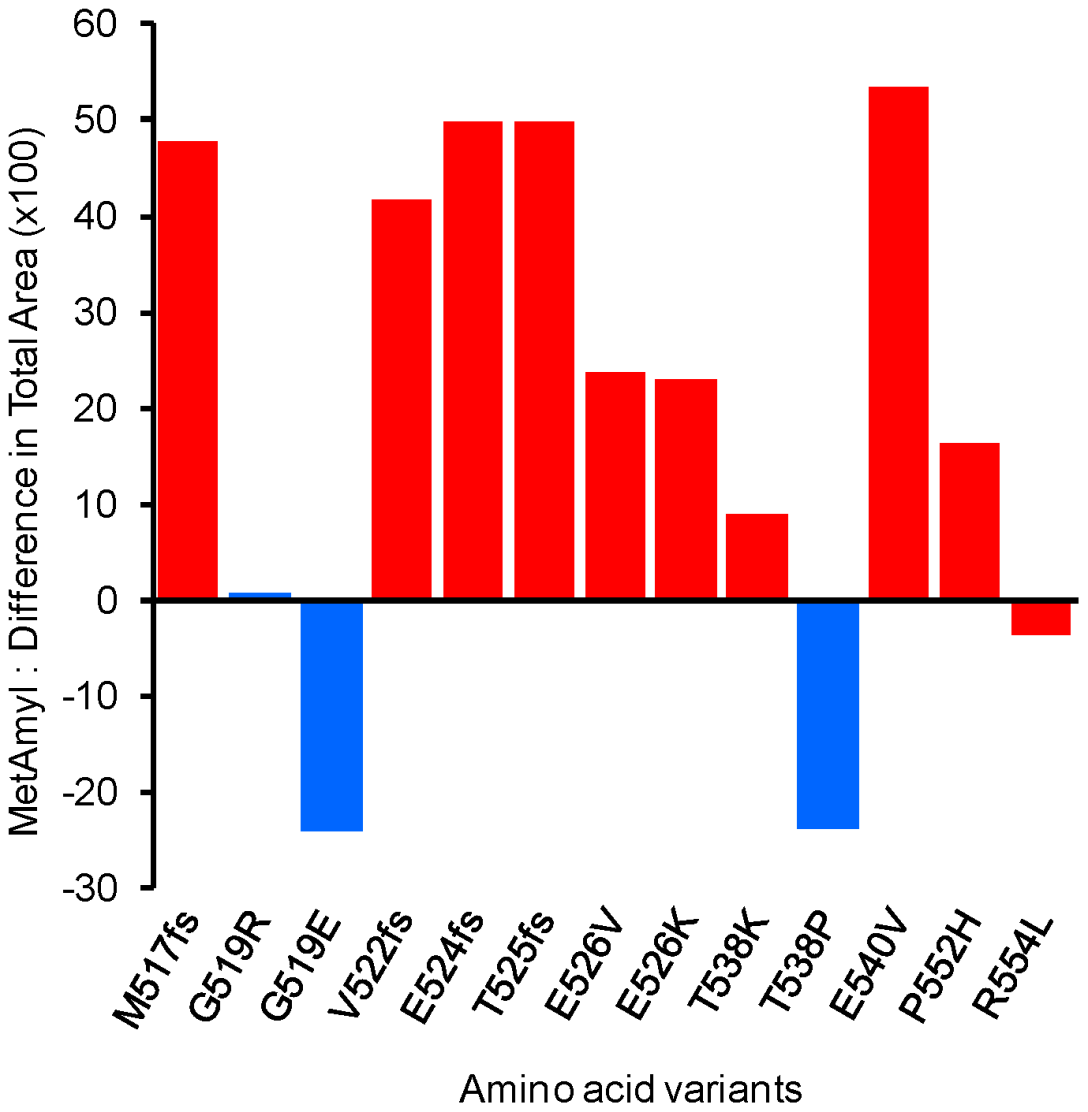
Metamyl predictions applied to human fibrinogen-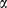. The effect of mutations is reported on a diagram where each column represents the difference of 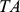 scores between the mutant and the corresponding wild-type sequence. The analysis is limited to mutations affecting the fragment of 80 amino acids found in amyloid fibrils, which is the region 500–580 of the mature protein. In red are variants involved in renal amyloidosis. In blue are non-pathological variants.

In this section, we assess the capacity for MetAmyl to predict the effect of single mutations in human fibrinogen-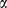. Fibrinogen has multiple biological functions and is a key protein of the coagulation pathway. Mutations in this gene lead to several disorders including hereditary renal/cardiac amyloidosis. The amyloid fibrils found in patients with renal amyloidosis are composed of fragments of fibrinogen encompassing residues 500 to 580 [51], [52]. So, we performed a systematic literature analysis in order to identify all known mutations affecting this region of the protein and linked to renal amyloidosis [53]–[61]. Our analysis also included screening of four databases: Ensembl [62], GEHT [63], cBioPortal [64], and www.amyloidosismutations.com. We compiled thirteen mutations having two types of consequences on fibrinogen: substitution of one amino-acid by another (missense mutation), or insertion of some amino-acids not found in the native sequence followed by premature termination of the protein (frameshift mutation). Only three missense mutations cause no detectable pathology, and are thus considered non-pathological variants. As shown in Figure 2, MetAmyl predicts how changes in the sequence of fibrinogen can affect its aggregation propensity. Moreover, one can see that 9 out of 10 mutations associated with renal amyloidosis show an increased of their 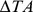 score.

## Discussion

By using a weighted combination of selected individual scores, MetAmyl has efficiently integrated predictors into a meta-predictor. We demonstrated in this paper the benefits of combining predictive algorithms based on a statistical framework. In the context of a complex trait, such as amyloidogenesis, merging existing predictors has allowed MetAmyl to account for a broad scale of features in a single predictor.

The evaluation of MetAmyl on three independent datasets revealed its accuracy to predict amyloidogenic segments in polypeptide chains and/or proteins. On the training dataset, MetAmyl has a significantly higher AUC, Accuracy and Matthews correlation coefficient than the other predictors. Moreover, on the amylome dataset, MetAmyl has the best Q value, Matthews correlation coefficient and F1 score. The potential overfitting for MetAmyl on the training dataset has been controlled by the use of cross-validation which is enhanced by MetAmyl performance on the amylome subset and the htt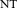 dataset. Although it is based on a large number of experimentally validated amyloid regions, the amylome subset suffers from a lack of validated non-amyloid regions, which can affect the results of performance calculations. For this reason, we used a third test set, named htt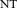 and independent of the training dataset the amylome subset. The htt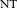 dataset has been chosen as being unbiased with regard to the correct assignment of amyloid or non-amyloid. Furthermore, the htt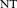 dataset illustrates the fact that polypeptides whose order has been randomly shuffled, can lead to various aggregation patterns (see Table S6). In the result section above, we showed that MetAmyl gives the best classification accuracy in comparison to 9 other predictors. Also, MetAmyl outperforms Zyggregator, software tested by the authors of the htt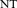 dataset [48], [65]. MetAmyl predicts a relevant hot spot of aggregation in four out of six amyloid-forming peptides (SP8, SP13, SP14, SP15). Moreover, none hot spot is predicted in the 10 non-amyloid peptides. The 100% specificity of MetAmyl on the htt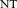 dataset is explained by a contribution from Waltz. In addition, FoldAmyloid correctly predicts the 6 amyloid-forming peptides, which greatly contributes to increase lack of sensitivity of Waltz. Waltz uses a position-specific scoring matrix for amyloid prediction, which, according to their authors, allows the distinction between amyloid fibrils and amorphous -sheet aggregates [29]. Perhaps it is a reason why peptides SP10 and SP11 are not correctly predicted by MetAmyl. Indeed, electron micrographs of scrambled sequences shown in [48] are far from representing well-ordered fibrillar structures.

MetAmyl behavior can be explained by the fact that it efficiently combines four complementary predictors: PAFIG, SALSA, Waltz and FoldAmyloid. First, our results demonstrated SALSA ability to detect amyloidogenic segments while being non-specific. SALSA has indeed a tendency to inappropriately predict hot spots: amyloidogenic prediction covers more than 56% of the studied subset of the amylome. Next, PAFIG and FoldAmyloid shared a very similar statistical pattern on both datasets. They are indeed sensitive (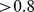) but not specific (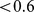) on the training dataset. Furthermore, PAFIG and FoldAmyloid are specific on the amylome subset (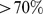) but lack in sensitivity especially FoldAmyloid (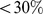). Finally, the Waltz predictor is very specific but lacks in sensitivity, which, in a sense, prevents MetAmyl from being too sensitive. Thus, MetAmyl can be seen as a trade-off between sensitive and specific methods leading to a significant improvement of the overall accuracy.

Moreover, combining PAFIG, SALSA, Waltz and FoldAmyloid allows MetAmyl to account for a large scale biological features related to amylose. In fact, MetAmyl prediction integrates the following features: the ability to form 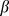-strands, the propensity to form 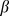-sheets [23], [25], disorder prediction [28], hydrophobicity [25], [29], structural modeling energy [29], support vector machine (SVM) exploited 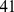 physico-chemical properties [25] and a position-specific matrix [29]. Our results confirm that all these features play a role in amyloidogenesis.

In figure 2 we show a remarkable example concerning the effects of disease-associated mutations on MetAmyl aggregation profiles. According to the algorithm, an amino acid substitution involves six contiguous hexapeptides, thus amplifying the change in the MetAmyl score in a sequence-dependent manner. As shown in the bar-graph, there is an increased 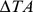 score in variants linked to renal amyloidosis, except for R554L. However, this variant was reported to be associated with dilated cardiomyopathy rather than restrictive cardiomyopathy typical of hereditary amyloidosis [58]. Two other variants at the same position in the sequence have been identified in patients with thrombosis [56], [59], [66]–[68]. The R554C variant has no renal deposits but shows an increase in the ability to self-associate (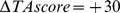). The clinical phenotype of the R554H variant is a thromboembolic pulmonary hypertension without renal amyloidosis (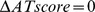). Although prediction of a clinical phenotype is not yet possible by bioinformatics methods, the observations done on fibrinogen suggest that MetAmyl can help to predict the effects of mutations on aggregation propensity of proteins. It should also be noted that such analysis cannot be performed by a predictor without a score of prediction such as AMYLPRED2 for example. The use of a quantitative score for MetAmyl allows for investigating the effect of variants.

Moreover, MetAmyl, available at http://metamyl.genouest.org/, allows for large-scale analysis of polypeptidic segments and/or proteins. The screening, with MetAmyl, of dedicated amyloid protein databases might help to better understand the formation of amyloid fibrils [67], . Thus, the propensity of some proteins to convert into their amyloid state might be investigated and MetAmyl could give new insights in explaining the development of neurodegenerative diseases.

## Conclusion

Accurate prediction of amyloid aggregation from the analysis of the primary sequence of proteins is a long and difficult way. In this paper we show that a statistical combination of several algorithms improves the reliability of individual methods. We sincerely thank all colleagues in the field who, in developing these methods have allowed the realization of an efficient meta-predictor. Generally, the predictors are tested on their ability to find amyloid-forming regions defined on the basis of in vitro experiments. However, it is known that amyloid fibers are polymorphic and their structural properties depend on many parameters [71]. Therefore, accurate prediction of amyloid-forming regions needs a large increase in properly validated benchmark datasets, without noise regarding classification in amyloid fibrils and amorphous 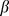-aggregates.

## Supporting Information

Figure S1 Receiver Operating Characteristic (ROC curves). ROC obtained for the 9 compared predictors on the training dataset. Predictor AMYLPRED2 is not plotted because it proposes a binary prediction which prevents the estimation of a ROC curve.
(TIFF)

Figure S2 Receiver Operating Characteristic (ROC curves). ROC obtained for the 9 compared predictors on the amylome subset. Predictor AMYLPRED2 is not plotted because it proposes a binary prediction which prevents the estimation of a ROC curve.
(TIFF)

Table S1 Variable selection steps. Values reported are Bayesian Information Criterion (BIC). * means that the variable is already in the model and the stepwise procedure tries to exclude it.
(PDF)

Table S2 Cross-validation for MetAmyl in the training dataset. Area under the curve (AUC), Accuracy (ACC) and Matthew's correlation coefficients (MCC) were computed for MetAmyl in four situation: No cross-validation (No CV), Leave-One-Out Cross-Validation (LOO), 10 Fold Cross-Validation (10Fold CV) and 20 Fold Cross-Validation (20Fold CV). Numbers in brackets correspond to 95% confidence intervals (95% C.I.) that were obtained using 2000 bootstrap replicates (Robin *et al*., 2011).
(PDF)

Table S3 Prediction performances based on the amylome dataset are given for the 10 compared predictors. For each method, the accuracy, the sensitivity, the specificity and the Matthews correlation coefficients (MCC) are reported. Numbers in brackets correspond to 95% confidence intervals (95% C.I.) that were obtained using bootstrap replicates (Robin *et al*., 2011).
(PDF)

Table S4 Area Under the Curve (AUC) based on the amylome subset. Area Under the Curve (AUC) was obtained from the ROC curves of 9 predictors: AUC cannot be computed for AMYLPRED2 as it provides only a binary prediction. For each method, the global AUC, the AUC for the False Positive Rate range of 0–20% and the AUC for the False Positive Rate range of 0–5% are reported. Numbers in brackets correspond to 95% confidence intervals (95% C.I.) that were obtained using bootstrap replicates (Robin *et al*., 2011). The comparison of MetAmyl AUC and the other method is summarized by the pvalue obtained with Delong's method (Delong *et al*., 1988).
(PDF)

Table S5 Prediction of amyloidogenic regions for MetAmyl on the Amylome subset. The residue numbering for the sequence features (second column) refers to the respective Uniprot entries. The residue numbering for the experimental and predicted regions (remaining columns) refers to the mature protein only. Sequences of the mature proteins as well as relevant literature used to obtain experimental information can be found in Table S1 of (Tsolis *et al*., 2013).
(PDF)

Table S6Descritption of the Huntingtin dataset. This table summarizes the experiments made by Roland *et al*. (2013) where amyloid forming properties have been studied for 16 sequences (SP1-SP15 and Htt
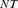
Q). Additionnal comments have been added for the sequences able to form amyloid fibrils. Furthermore, MetAmyl hot spots prediction is given in the last column.(PDF)

Table S7 Prediction performances based on the Huntingtin dataset are given for the 10 compared predictors. For each method, the accuracy, the sensitivity, the specificity and the Matthews correlation coefficients (MCC) are reported. Numbers in brackets correspond to 95% confidence intervals (95% C.I.) that were obtained using bootstrap replicates (Robin *et al.*, 2011).
(PDF)

Text S1 Text for supporting information.
(PDF)
