# Supplementary figures and images for: MetAmyl: A METa-Predictor for AMYLoid Proteins
Source: PLoS One. 2013 Nov 19;8(11):e79722. doi: 10.1371/journal.pone.0079722 (PMC3834037; doi:10.1371/journal.pone.0079722)

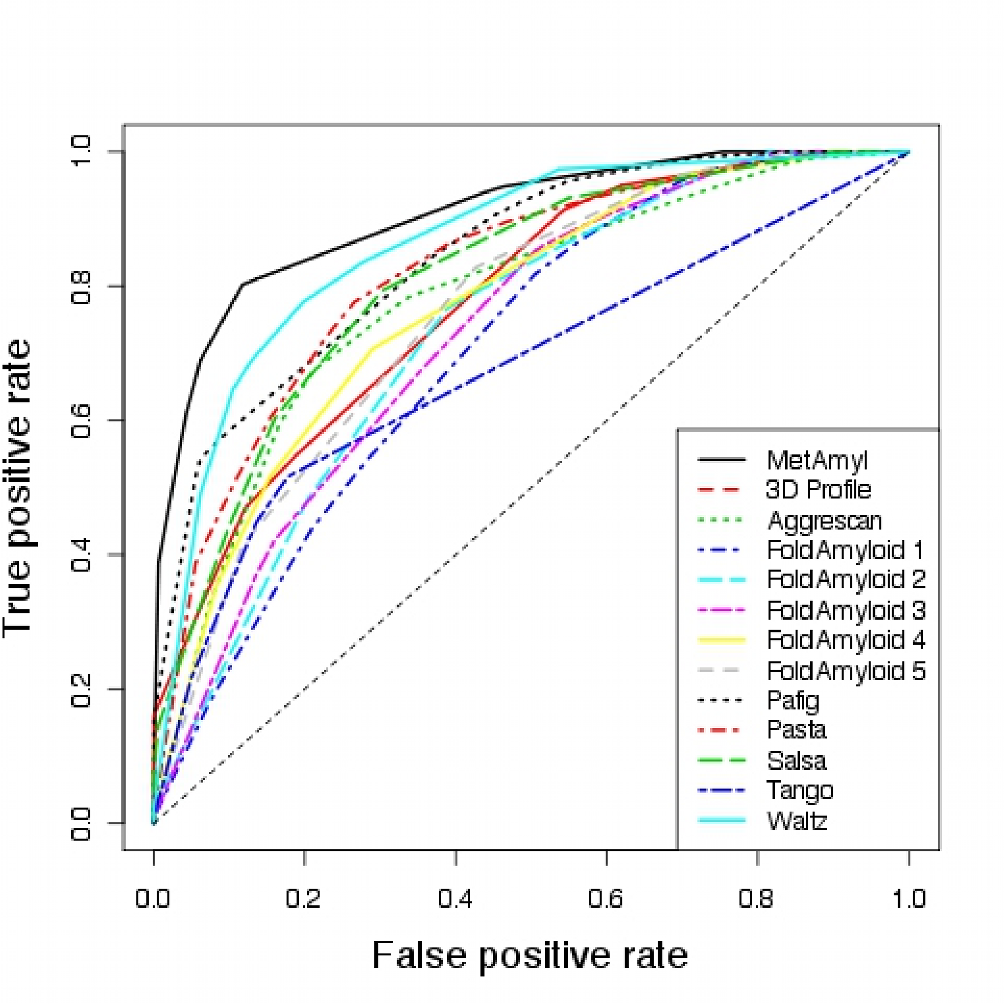

Supplement: Figure S1 — Receiver Operating Characteristic (ROC curves). ROC obtained for the 9 compared predictors on the training dataset. Predictor AMYLPRED2 is not plotted because it proposes a binary prediction which prevents the estimation of a ROC curve. (TIFF) [file pone.0079722.s001.tiff]

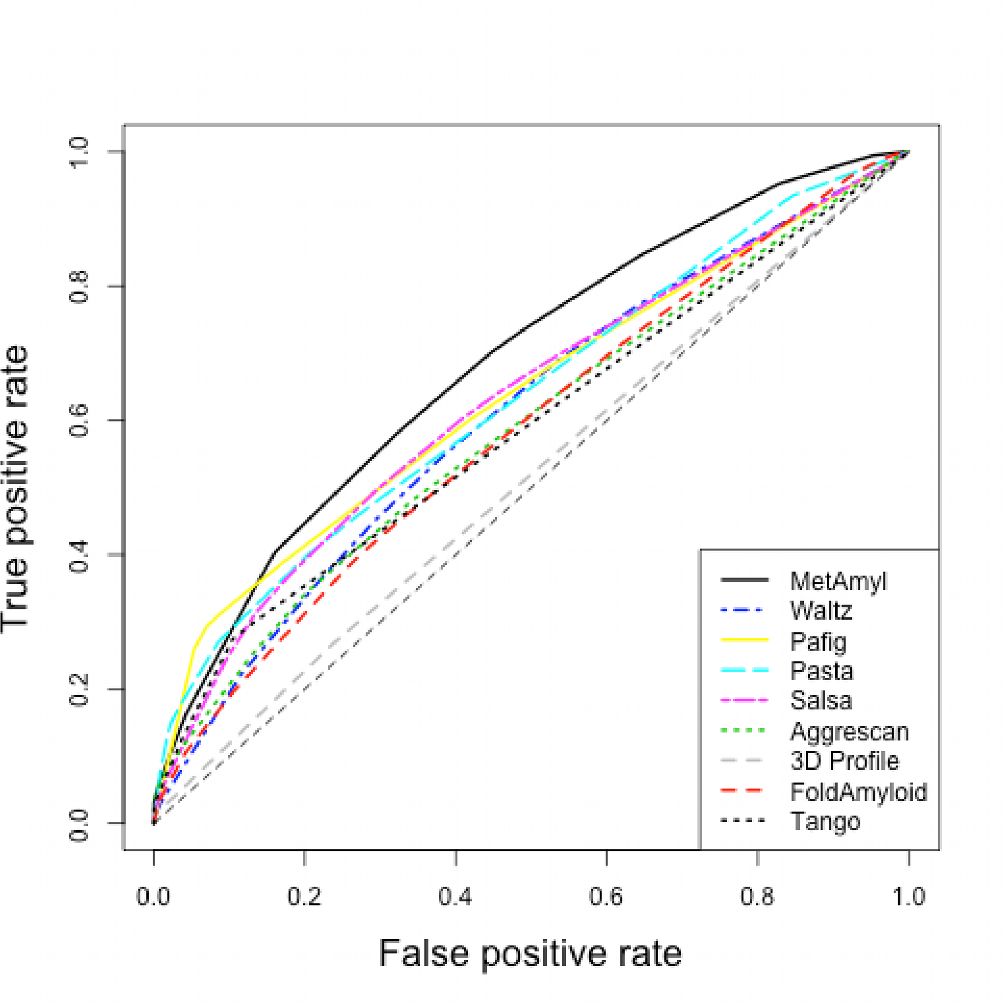

Supplement: Figure S2 — Receiver Operating Characteristic (ROC curves). ROC obtained for the 9 compared predictors on the amylome subset. Predictor AMYLPRED2 is not plotted because it proposes a binary prediction which prevents the estimation of a ROC curve. (TIFF) [file pone.0079722.s002.tiff]
