# Supplementary material for: MetAmyl: A METa-Predictor for AMYLoid Proteins
Source: PLoS One. 2013 Nov 19;8(11):e79722. doi: 10.1371/journal.pone.0079722 (PMC3834037; doi:10.1371/journal.pone.0079722)
Supplement: Table S1 — Variable selection steps. Values reported are Bayesian Information Criterion (BIC). * means that the variable is already in the model and the stepwise procedure tries to exclude it. (PDF) [file pone.0079722.s003.pdf]

Step	Current BIC	AGGRESCAN	FA1	FA2	FA3	FA4	FA5	PAFIG	SALSA	PASTA	TANGO	Waltz
1	383.3708	337.31	362.9806	348.0414	353.3039	336.3962	344.5257	**297.0727**	312.8624	310.6046	369.0856	333.3373
2	297.0727	288.4717	297.6191	290.2835	293.805	285.3141	290.2659	383.3708*	**265.6044**	274.2781	295.9853	273.0383
3	265.6044	265.9601	266.4827	271.1545	269.9517	270.7621	270.1404	312.8624*	297.0727*	271.2072	270.7364	**252.1665**
4	252.1665	252.8436	**250.6343**	257.6457	254.6721	256.848	255.2327	285.8381*	273.0383*	257.5605	257.2166	265.6044*
5	**250.6343**	256.2219	252.1665*	252.7962	252.7827	255.7218	254.3185	289.5859*	277.1560*	255.7986	255.8080	266.4827*
