# Supplementary material for: MetAmyl: A METa-Predictor for AMYLoid Proteins
Source: PLoS One. 2013 Nov 19;8(11):e79722. doi: 10.1371/journal.pone.0079722 (PMC3834037; doi:10.1371/journal.pone.0079722)
Supplement: Table S2 — Cross-validation for MetAmyl in the training dataset. Area under the curve (AUC), Accuracy (ACC) and Matthew's correlation coefficients (MCC) were computed for MetAmyl in four situation: No cross-validation (No CV), Leave-One-Out Cross-Validation (LOO), 10 Fold Cross-Validation (10Fold CV) and 20 Fold Cross-Validation (20Fold CV). Numbers in brackets correspond to 95% confidence intervals (95% C.I.) that were obtained using 2000 bootstrap replicates (Robin et al., 2011). (PDF) [file pone.0079722.s004.pdf]

Predictor	AUC [95% CI]	ACC [95% C.I.]	MCC [95% C.I.]
MetAmyl No CV	0.91 [0.88-0.94]	0.85 [0.83-0.89]	0.70 [0.65-0.76]
MetAmyl LOO CV	0.89 [0.87-0.92]	0.84 [0.81-0.87]	0.67 [0.60-0.72]
MetAmyl 10Fold CV	0.89 [0.86-0.92]	0.84 [0.80-0.87]	0.67 [0.59-0.70]
MetAmyl 20Fold CV	0.89 [0.87-0.93]	0.84 [0.81-0.87]	0.67 [0.59-0.71]
