# Supplementary material for: MetAmyl: A METa-Predictor for AMYLoid Proteins
Source: PLoS One. 2013 Nov 19;8(11):e79722. doi: 10.1371/journal.pone.0079722 (PMC3834037; doi:10.1371/journal.pone.0079722)
Supplement: Table S3 — Prediction performances based on the amylome dataset are given for the 10 compared predictors. For each method, the accuracy, the sensitivity, the specificity and the Matthews correlation coefficients (MCC) are reported. Numbers in brackets correspond to 95% confidence intervals (95% C.I.) that were obtained using bootstrap replicates (Robin et al., 2011). (PDF) [file pone.0079722.s005.pdf]

Predictor	Q [95% CI]	MCC [95% CI]	F1 [95% CI]
MetAmyl	62.27 [61.32-63.21]	0.23 [0.21-0.24]	0.36 [0.35-0.37]
Waltz	60.93 [59.95-61.78]	0.16 [0.15-0.17]	0.33 [0.32-0.34]
PAFIG	61.59 [60.64-62.45]	0.18 [0.16-0.19]	0.34 [0.33-0.35]
PASTA	55.54 [54.63-56.51]	0.14 [0.13-0.15]	0.23 [0.22-0.24]
SALSA	58.54 [57.67-59.51]	0.13 [0.12-0.14]	0.31 [0.30-0.32]
AGGRESCAN	57.32 [56.42-58.26]	0.13 [0.12-0.14]	0.29 [0.28-0.30]
3D profile	52.74 [51.97-53.73]	0.06 [0.05-0.07]	0.20 [0.19-0.21]
FoldAmyloid	56.60 [55.71-57.55]	0.13 [0.12-0.14]	0.27 [0.26-0.28]
TANGO	54.62 [53.61-55.53]	0.14 [0.13-0.15]	0.20 [0.19-0.21]
AMYLPRED2	61.02 [60.10-61.97]	0.20 [0.18-0.21]	0.34 [0.33-0.35]
