# Supplementary material for: MetAmyl: A METa-Predictor for AMYLoid Proteins
Source: PLoS One. 2013 Nov 19;8(11):e79722. doi: 10.1371/journal.pone.0079722 (PMC3834037; doi:10.1371/journal.pone.0079722)
Supplement: Table S4 — Area Under the Curve (AUC) based on the amylome subset. Area Under the Curve (AUC) was obtained from the ROC curves of 9 predictors: AUC cannot be computed for AMYLPRED2 as it provides only a binary prediction. For each method, the global AUC, the AUC for the False Positive Rate range of 0–20% and the AUC for the False Positive Rate range of 0–5% are reported. Numbers in brackets correspond to 95% confidence intervals (95% C.I.) that were obtained using bootstrap replicates (Robin et al., 2011). The comparison of MetAmyl AUC and the other method is summarized by the pvalue obtained with Delong's method (Delong et al., 1988). (PDF) [file pone.0079722.s006.pdf]

Predictor	AUC [95% CI]	pvalue (AUC vs MetAmyl AUC)	AUC [95% CI] (FPR: 0-20%)	AUC [95% CI] (FPR: 0-5%)
MetAmyl	0.67 [0.66-0.69]	1	0.052 [0.050-0.055]	0.0046 [0.0040-0.0052]
Waltz	0.60 [0.58-0.62]	9.3×10^{-12}	0.035 [0.032-0.037]	0.0024 [0.0017-0.0030]
PAFIG	0.62 [0.61-0.64]	4.3×10^{-7}	0.054 [0.051-0.056]	0.0032 [0.0026-0.0038]
PASTA	0.61 [0.59-0.63]	3.3×10^{-11}	0.052 [0.050-0.055]	0.0051 [0.0040-0.0052]
SALSA	0.61 [0.60-0.64]	3.2×10^{-12}	0.046 [0.044-0.048]	0.0039 [0.0033-0.0045]
AGGRESCAN	0.57 [0.55-0.59]	$< 2.2 \times 10^{-16}$	0.039 [0.036-0.041]	0.0042 [0.0036-0.0047]
3D profile	0.53 [0.52-0.55]	$< 2.2 \times 10^{-16}$	0.028 [0.025-0.030]	0.0022 [0.0036-0.0047]
FoldAmyloid	0.58 [0.56-0.60]	$< 2.2 \times 10^{-16}$	0.035 [0.033-0.037]	0.0032 [0.0026-0.0038]
TANGO	0.58 [0.57-0.60]	$< 2.2 \times 10^{-16}$	0.030 [0.028-0.032]	0.0039 [0.0033-0.0045]
