# Supplementary material for: MetAmyl: A METa-Predictor for AMYLoid Proteins
Source: PLoS One. 2013 Nov 19;8(11):e79722. doi: 10.1371/journal.pone.0079722 (PMC3834037; doi:10.1371/journal.pone.0079722)
Supplement: Table S5 — Prediction of amyloidogenic regions for MetAmyl on the Amylome subset. The residue numbering for the sequence features (second column) refers to the respective Uniprot entries. The residue numbering for the experimental and predicted regions (remaining columns) refers to the mature protein only. Sequences of the mature proteins as well as relevant literature used to obtain experimental information can be found in Table S1 of (Tsolis et al., 2013). (PDF) [file pone.0079722.s007.pdf]

Protein name	UniProtKB Ac	Sequence length	Experimental regions		MetAmyl hot spots	
			Begin-End	Sequence	Begin-End	Sequence
Acylphosphatase-2	P14621	98	16-31<br>87-98	RVQGVCFRMYTEDEAR<br>SKLEYSNFSIRY	17-22<br>83-88<br>31-53	VQGVCF<br>RKIGVVGWVKNTSKGTVTGQVQG<br>EKTISK
Amphoterin (Rat)	P63159	214	12-27	MSSYAFFVQTCREEHK	14-22<br>33-38<br>173-178	SYAFFVQTC<br>ASVNFS<br>GVVKAE
IAPP (Amylin)	P10997	37	08-20<br>14-20<br>20-29<br>21-30<br>30-37	ATQRLANFLVHSS<br>NFLVHSS<br>SNNFGAILSS<br>NNFGAILSST<br>TNVGSNTY	14-22<br>24-32	NFLVHSSNN<br>GAILSSTNV
Apolipoprotein A-I	P02647	243	01-93	DEPPQSPWDRVKDLATVYVDVLKDSGRDYVSQFEGSA<br>LGKQLNLKLLDNWDSVTSTFSKLREQLGPVTQEFWDN<br>LEKETEGLRQEMSKDLEEV	14-24<br>30-35<br>49-60<br>63-72<br>225-232	LATVYVDVLKD<br>VSQFEG<br>NWDSVTSTFSKL<br>QLGPVTQEFW<br>FKVSFLSA
Apolipoprotein C-II	P02655	79	60-70	MSTYTGIFTDQ	13-21<br>61-76	TFLTQVKES<br>STYTGIFTDQVLSVLK
Amyloid beta A4 protein	P05067	42	11-25<br>25-35<br>30-40<br>37-42	EVHHQKLVFFAEDVG<br>GSNKGAIIGLM<br>AIIGLMVGGVV<br>GGVVIA	14-21<br>31-42	HQKLVFFA<br>IIGLMVGGVVIA
beta2-Microglobulin	P61769	99	21-31<br>33-41<br>59-71<br>83-89<br>91-96	NFLNCYVSGFH<br>SDIEVDLLK<br>DWSFYLLYYTEFT<br>NHVTLSQ<br>KIVKWD	22-32<br>68-73<br>83-89<br>91-96	FLNCYVSGFHP<br>TEFTPT<br>NHVTLSQ<br>KIVKWD
Beta-lactoglobulin	P02754	162	11-20<br>101-110<br>116-126<br>146-152	DIQKVAGTWY<br>KYLLFCMENS<br>SLACQCLVRTP<br>HIRLSFN	01-07<br>80-85<br>91-97	LIVTQTM<br>AVFKID<br>KVLVLDT
Calcitonin	P01258	32	15-19<br>15-20	DFNKF<br>DFNKFH	24-30	QTAIGVG
Casein	P02663	207	81-125	ALNEINQFYQKFPQYLQYLYQGPIVLNPWDQVKRNAV<br>PITPTLNR	34-39<br>69-74<br>96-101<br>134-139<br>179-186<br>199-205	NLCSTF<br>VKITVD<br>LQYLYQ<br>NSKKTV<br>YLKTVYQH<br>KVIPYVR
Cold shock protein	P32081	67	01-22<br>01-35<br><br>36-67	MLEGKVKWFNSEKGFGFIEVEG<br>MLEGKVKWFNSEKGFGFIEVEGQDDVFVHFSAIQG<br>EGFKTLEEGQAVSFEIVEGNRGPQAANVTKEA	26-34<br>44-52	VFVHFSAIQ<br>GQAVSFEIV
Cystatin C	P01034	120	98-103	SFQIYA	55-64<br>97-104	QIVAGVNYFL<br>CSFQIYAV
Myoglobin (Horse)	P68082	153	01-29<br>101-118	GLSDGEWQQVLNVWGKVEADIAGHGQEVL<br>IKYLEFISDAIIHVLHSK	08-18<br>67-72	QQVLNVWGKVE<br>VVLTAL
Gelsolin	P06396	755	173-230	ATEVPVSWESFNNGDCFILDLGNNIHQWCGSNSNRYE<br>RLKATQVSKGIRDNERSGRAR	67-74<br>101-108<br>122-127<br>142-162<br>186-191<br>212-218<br>283-288<br>312-317<br>347-353<br>445-452<br>460-465<br>481-486<br>495-500<br>536-541<br>566-571<br>579-584<br>650-655<br>679-685<br>715-724	AYVILKTV<br>AAIFTVQL<br>VQGFES<br>KGGVASGFKHVVPNEVVVQRL<br>GDCFIL<br>KATQVSK<br>MSVSLV<br>DGKIFV<br>QTQVSVL<br>DSYIILYN<br>GQIIYN<br>ILTAQL<br>VQSRVV<br>TRLFQV<br>AFVLKT<br>VGTGAS<br>GRFVIE<br>QVFVWVG<br>TPITVVKQGF
Het-s	Q03689	289	218-289	KIDAIVGRNSAKDIRTEERARVQLGNVVTAAALHGGIRI<br>SDQTTNSVETVVGKGESRVLIGNEYGGKGFWDN	05-10<br>25-30<br>79-87<br>108-113<br>159-164<br>195-200<br>219-224<br>239-249<br>259-278	FGIVAG<br>FEYVQL<br>LAKSIVEEI<br>QDLVVF<br>IVDQVA<br>ASLTIL<br>IDAIVG<br>VQLGNVVTAAA<br>QTTNSVETVVGKGESRVLIG
InsulinA	P01308	21	13-18	LYQLEN	02-15	IVEQCCTSICSLYQ
InsulinB	P01308	30	11-17	LVEALYL	07-12	CGSHLV

Kerato-epithelin	Q15582	660	492-502<br>492-509	FSMLVAAIQSA<br>FSMLVAAIQSAGLTETLN	24-33<br>50-61<br>86-99<br>113-118<br>132-142<br>184-192<br>201-217<br>220-225<br>298-304<br>336-345<br>363-368<br>440-445<br>482-487<br>492-500<br>511-520<br>554-564<br>580-587<br>597-610	NVCAVQKVIG<br>ICGKSTVISYEC<br>TLGVVGSTTTQLYT<br>GSFTIF<br>VLDSLVSNVNI<br>NGIVTVNCA<br>ATNGVVHLIDKVISTIT<br>IQQIIE<br>IVAGLSV<br>ATNGVIHYID<br>SDVSTA<br>LRVFVY<br>VMDVLK<br>FSMLVAAIQ<br>EGVYTVFAPT<br>ILVSGGIGALV<br>NNVVSVNK<br>ATNGVVHVITNVLQ
Lactoferrin	P02788	691	538-545	NAGDVAFV	06-11<br>61-66<br>92-100<br>153-158<br>204-209<br>255-260<br>285-290<br>305-310<br>323-329<br>345-350<br>366-377<br>399-404<br>435-442<br>449-454<br>540-554<br>663-673	VQWCAV<br>GGFIYE<br>YYAVAVVKK<br>FSASCV<br>GDVAFI<br>VVARSV<br>KFQLFG<br>IGFSRV<br>GYFTAIQ<br>VVWCAV<br>EGSVTCSSASTT<br>VYTAGK<br>YLAVAVVR<br>TWNSVK<br>GDVAFVKDVTVLQNT<br>LGPQYVAGITN
Lung Surfactant	P11686	35	09-34	HLKRLLIVVVVVVLVVVVIVGALLMG	03-08<br>12-32	IPCCPV<br>RLLIVVVVVVLVVVVIVGALL
Lysozyme C	P61626	130	05-14<br>25-34<br>56-61	RCELARTLKR<br>LANWMCLAKW<br>IFQINS	123-128	CQYVQGC
Major Prion Protein	P04156	231	84-104<br>91-105<br>105-125<br>116-122<br>148-153<br>154-163<br>156-171<br>180-196<br>209-231	KTNMKHMAGAAAAGAVVGGLG<br>AGAAAAGAVVGGLGG<br>GYMLGSAMSRPIIHFGSDYED<br>IIHFGSD<br>SNQNNF<br>VHDCVNITIK<br>DCVNITIKQHTVTTTT<br>DVKMMERVVEQMCITQY<br>SMVLFSSPPVILLISFLIFLIVG	95-104<br>112-121<br>137-142<br>154-171<br>184-192<br>208-231	AAGAVVGGLG<br>MSRPIIHFGS<br>NQVYYR<br>VHDCVNITIKQHTVTTTT<br>MERVVEQMC<br>SSMVLFSSPPVILLISFLIFLIVG
Medin	Q08431	50	32-41<br>42-50	VTGIITQGAR<br>NFGSVQFVA	12-17<br>31-38<br>42-50	VAGSYG<br>EVTGIITQ<br>NFGSVQFVA
proBNP	P16860	108	66-72	KMVLYTL	48-53<br>66-71	TGVWKS<br>KMVLYT
ODAM	A1E959	264	112-157	MPYVFSFKMPQEQGQMFQYYPVYMVLPWEQPQQT<br>VPRSPQQTRQQQ	127-136<br>234-243	MFQYYPVYMV<br>STTNVFTSAV
Prolactin	P01236	199	07-21<br>20-34<br>43-57	GAARCQVTLRDLFDR<br>DRAVVLSHYIHNLSS<br>RYTHGRGFITKAINS	24-29<br>51-56<br>80-90<br>98-103<br>112-117<br>129-138	VLSHYI<br>ITKAIN<br>FLSLIVSILRS<br>LVTEVR<br>ILSKAV<br>GMELIVSQVH
RepA	Q52546	209	05-13	LVLCAASLI	35-41<br>63-68<br>74-79	VFGIDVK<br>VKGKVV<br>VFHVKY
Semenogelin I	P04279	439	1-142	QKGGSKGRLPSEFSQFPHGQKGQHYSGQKGKQQTES<br>KGSFSIQYTYHVDANDHDQSRKSQQYDLNALHKTTKS<br>QRHLGGSQQLLHNKQEGRDHDKSKGHFHRVVIHHKG<br>GKAHRGTQNPSQDQGNSPSGKGISSQYSNTEER	40-48<br>100-105<br>150-155<br>167-175<br>196-205<br>211-216<br>358-363<br>410-418	FSIQYTYHV<br>FHRVVI<br>KEQTSV<br>SQSSYVLQT<br>HYQNVVEVRE<br>VQTSLC<br>LVAGKS<br>GLDIVIIEQ
Serum Amyloid A	P02735	104	01-12	RSFFSFLGEAFD	02-07	SFFSFL

Sup35	P05453	685	07-13	GNNQQNY	09-14<br>31-36<br>45-50<br>143-148<br>259-266<br>363-372<br>397-408<br>412-417<br>422-431<br>438-447<br>529-536<br>566-572<br>578-591<br>597-610<br>612-617<br>639-648<br>651-658<br>671-684	NQQNYQ<br>GYQAYN<br>YYQNYQ<br>TLKLVS<br>DHVSLIFM<br>DVGVLVISAR<br>GVNKMVVVVNKM<br>TVNWSK<br>QCVSNVSNFL<br>IKTDVVFMPV<br>VEIQNIYN<br>GFVLTSP<br>SVTKFVAQIAIVEL<br>AGFSCVMHVHTAIE<br>VHIVKL<br>GMKVIAVLET<br>PVCVETYQ<br>QGTTIAIGKIVKIA
alpha-Synuclein	P37840	140	35-44<br>49-59<br>60-68<br>69-82<br>86-95	EGVLYVGSKT<br>VHGVATVAEKT<br>KEQVTNVGG<br>AVVTGVTAVAQKTV<br>GSIAAATGFV	11-19<br>35-86<br><br><br>90-96	AKEGVVAAA<br>EGVLYVGSKTKEGVVHGVATVAEKTKEQVTNVGGAVV<br>TGVTAVAQKTVEGAG<br>AATGFVK
Tau	P10636	757	589-600	GKVQIINKKLDL	118-123<br>270-276<br>301-307<br>440-446<br>540-547<br>588-596<br>603-608<br>620-641<br>714-719<br>723-730	AGHVTQ<br>FLSKVST<br>TFHVEIT<br>HVSSVTS<br>KKVAVVRT<br>GGKVQIINK<br>VQSKCG<br>GSVQIVYKPVDLSKVTSKCGSL<br>VVSGDT<br>HLSNVSST
Transthyretin	P02766	127	10-20<br>105-115	CPLMVKVLDAV<br>YTIAALLSPYS	11-20	PLMVKVLDAV
Ure2p	P23202	354	01-89<br><br><br>10-39	MMNNNGNQVSNLSNALRQVNIGNRNSNTTTDQSNI<br>NFEFSTGVNNNNNNNSSSNNNNVQNNNSGRNGSQ<br>NNDNENNIKNTLEQHRQQQQ<br>SNLSNALRQVNIGNRNSNTTTDQSNINFEF	39-44<br>126-135<br>138-144<br>155-160<br>213-218<br>240-245<br>254-260<br>309-314	FSTGVN<br>FKVAIVLSEL<br>HYNTIFL<br>EFVSVN<br>NAWLFF<br>KIASAV<br>RVYGVVE<br>ADLAFV
