# Supplementary material for: MetAmyl: A METa-Predictor for AMYLoid Proteins
Source: PLoS One. 2013 Nov 19;8(11):e79722. doi: 10.1371/journal.pone.0079722 (PMC3834037; doi:10.1371/journal.pone.0079722)
Supplement: Table S6 — Descritption of the Huntingtin dataset. This table summarizes the experiments made by Roland et al. (2013) where amyloid forming properties have been studied for 16 sequences (SP1-SP15 and HttQ). Additionnal comments have been added for the sequences able to form amyloid fibrils. Furthermore, MetAmyl hot spots prediction is given in the last column. (PDF) [file pone.0079722.s008.pdf]

Name	Sequence	Amyloid forming peptide	Comments	MetAmyl hot spots
SP1	TMMKFQLLKSAEEKLFAS	No		
SP2	MLSLKESAKMFFATKELQ	No		
SP3	KQFTLEMAFLSKALSEMK	No		
SP4	KLAFMLKQAELSSEKTFM	No		
SP5	FAKFASEKKLESMTLMLQ	No		
SP6	MLTFAEFKSMELKSQLAK	No		
SP7	ASMFEAQLSKEKKMFTLL	No		
SP8	ELLAKSEQAKSMLFTFMK	Yes	Amyloid aggregation only at high concentration	KSMLFT
SP9	TKFSSFALLAQKEMLKME	No		
SP10	ETLKMSMFLEAQFKKSAL	Yes	Fast and high amyloid aggregation kinetic	
SP11	ASSQKKMKEMLAFFTLEL	Yes	Slow amyloid aggregation kinetic	
SP12	MFSKMAKSLFLLAEKTQE	No		
SP13	KLELKAASQMEFSFTMKL	Yes	Slow amyloid aggregation kinetic	SFTMKL
SP14	KELKQELFFKASATLMMS	Yes	Fast and high amyloid aggregation kinetic	KQELFF
SP15	SAFMEKMLLLEKQFKAST	Yes	Fast and high amyloid aggregation kinetic	QFKAST
$Htt^{NT}Q$	MATLEKLMKAFESLKSFQ	No	Amorphous aggregates (α-helix-rich oligomers)	
