# Supplementary material for: MetAmyl: A METa-Predictor for AMYLoid Proteins
Source: PLoS One. 2013 Nov 19;8(11):e79722. doi: 10.1371/journal.pone.0079722 (PMC3834037; doi:10.1371/journal.pone.0079722)
Supplement: Table S7 — Prediction performances based on the Huntingtin dataset are given for the 10 compared predictors. For each method, the accuracy, the sensitivity, the specificity and the Matthews correlation coefficients (MCC) are reported. Numbers in brackets correspond to 95% confidence intervals (95% C.I.) that were obtained using bootstrap replicates (Robin et al., 2011). (PDF) [file pone.0079722.s009.pdf]

Predictor	Q [95% CI]	MCC [95% CI]	F1 [95% CI]
MetAmyl	83.33 [75.81-100]	0.75 [0.54-1.00]	0.80 [0.67-1.00]
Waltz	66.67 [50.01-74.78]	0.48 [0.10-0.79]	0.50 [0.40-0.67]
PAFIG	56.67 [44.64-71.43]	0.15 [-0.16-0.50]	0.55 [0.50-0.71]
PASTA	51.67 [39.58-64.29]	0.04 [-0.29-0.39]	0.52 [0.46-0.67]
SALSA	71.67 [58.33-85.71]	0.42 [0.13-0.74]	0.66 [0.60-0.80]
AGGRESCAN	55.00 [50.01-58.33]	0.20 [0-0.37]	0.57 [0.46-0.70]
3D profile	33.33 [24.83-50.01]	-0.49 [-0.71-0]	0.40 [0.30-0.53]
FoldAmyloid	55.00 [50.01-58.33]	0.20 [0-0.37]	0.57 [0.46-0.70]
TANGO	51.67 [35.03-67.86]	0.03 [-0.32-0.49]	0.36 [0.25-0.57]
AMYLPRED2	75.00 [64.29-83.33]	0.52 [0.33-0.76]	0.70 [0.60-0.83]
