# Supplementary material for: MetAmyl: A METa-Predictor for AMYLoid Proteins
Source: PLoS One. 2013 Nov 19;8(11):e79722. doi: 10.1371/journal.pone.0079722 (PMC3834037; doi:10.1371/journal.pone.0079722)
Supplement: Text S1 — Text for supporting information. (PDF) [file pone.0079722.s010.pdf]

Supporting information for "MetAmyl: a METa-predictor for AMYLoid proteins"

Mathieu Emily, Anthony Talvas, Christian Delamarche

Supplementary Methods

Stepwise variable selection. The selection of the four individual predictors (PAFIG, SALSA, Waltz and FoldAmyloid) has been made by a stepwise regression algorithm that aims at selecting iteratively the most informative predictor to be included in the current model. In MetAmyl, we used a logistic regression model, and variable selection was based on the Bayesian Informative Criterion (BIC). Basically, at each step, the predictor for which the incorporation in the model provides the biggest decrease in the BIC is considered as the most informative predictor and is included in the model in the next step. In the stepwise selection algorithm, the addition of a new individual predictor is followed by the elimination of any predictor that does not provide any significant information in the model anymore.

Numerical details of each step is provided in Table S1. The selection starts with a model with no predictor, $\mathscr{M}_0$, that have a BIC of 383.3708.

$$\mathscr{M}_0 : \quad \log\left(\frac{p(x)}{1-p(x)}\right) = \beta_0, \quad BIC = 383.3708$$

The most informative predictor is PAFIG (lower BIC in the first row of Table S1 so after the first step the current model is $\mathscr{M}_1$ given by:

$$\mathscr{M}_1 : \quad \log\left(\frac{p(x)}{1-p(x)}\right) = \beta_0 + \beta_1 S_{PAFIG}(x), \quad BIC = 297.0727$$

At this step, the most informative additional predictor is SALSA (lower BIC in the second row of Table S1. The current model becomes model $\mathscr{M}_2$ given by:

$$\mathscr{M}_2 : \quad \log\left(\frac{p(x)}{1-p(x)}\right) = \beta_0 + \beta_1 S_{PAFIG}(x) + \beta_2 S_{SALSA}(x), \quad BIC = 265.6044$$

The next predictor included in the model is Waltz (lower BIC in the third row of Table S1. The model becomes model $\mathscr{M}_3$ given by:

$$\mathscr{M}_3 : \quad \log\left(\frac{p(x)}{1-p(x)}\right) = \beta_0 + \beta_1 S_{PAFIG}(x) + \beta_2 S_{SALSA}(x) + \beta_3 S_{Waltz}(x), \quad BIC = 252.1665$$

The last predictor included in the model is FoldAmyloid (lower BIC in the fourth row of Table S1. Current model becomes $\mathscr{M}_4$ given by:

$$\mathscr{M}_4 : \quad \log\left(\frac{p(x)}{1-p(x)}\right) = \beta_0 + \beta_1 S_{PAFIG}(x) + \beta_2 S_{SALSA}(x) + \beta_3 S_{Waltz}(x) + \beta_4 S_{FA1}(x), \quad BIC = 250.6343$$

The stepwise procedure stops at the fifth step as the inclusion of a new predictor in the model $\mathscr{M}_4$ results in an increase of the BIC. Thus, none of non-included predictor give significant additional information. In the fifth row of Table S1, all BIC are higher than the current BIC.

MetAmyl score. The MetAmyl logistic regression model is defined as follows:

$$\log\left(\frac{p(x)}{1-p(x)}\right) = \beta_0 + \beta_1 S_{PAFIG}(x) + \beta_2 S_{SALSA}(x) + \beta_3 S_{Waltz}(x) + \beta_4 S_{FA1}(x)$$

where

$\beta_0 = -0.047727784$, $\beta_1 = 3.667188941$, $\beta_2 = 4.944766967$, $\beta_3 = 0.005114034$, $\beta_4 = -0.413373395$

Interpretation of the coefficients. In logistic regression framework, β-coefficients can be interpreted in terms of odds-ratio. For example, coefficient $\beta_3 = 0.0005114034$, that weights Waltz score, means that an increase of 1 in the Waltz score results in a multiplication of the odds by a factor of $\exp(\beta_3)$, assuming that all other scores remain the same, (more details can be found in the following reference : Nelder, John; Wedderburn, Robert, 1972, “Generalized Linear Models”. Journal of the Royal Statistical Society. Series A). As a consequence, the interpretation of the value of the β coefficients depends on the scale of each individual score and more specifically on their variance or standard deviation. In the training dataset, standard deviations for Pafig, Salsa, Waltz and FA1 scores were respectively given by 0.33, 0.27, 158 and 1.43. One can remark that the absolute value of the β-coefficient is inversely proportional to the standard deviation which indicates that PAFIG, SALSA, Waltz and FA1 provide equivalent contribution to MetAmyl score.
